# Polyphenol-rich *Avicennia marina* leaf extracts induce apoptosis in human breast and liver cancer cells and in a nude mouse xenograft model

**DOI:** 10.18632/oncotarget.8624

**Published:** 2016-04-07

**Authors:** Cheng Huang, Chung-Kuang Lu, Ming-Chin Tu, Jia-Hua Chang, Yen-Ju Chen, Yu-Hsuan Tu, Hsiu-Chen Huang

**Affiliations:** ^1^ National Research Institute of Chinese Medicine, Ministry of Health and Welfare, Taipei 11221, Taiwan; ^2^ Department of Life Sciences and Institute of Genome Sciences, National Yang-Ming University, Taipei 11221, Taiwan; ^3^ Department of Applied Science, National Hsinchu University of Education, Hsinchu 30014, Taiwan

**Keywords:** Avicennia marina, xenografts, MDA-MB-231, HepG2, AU565

## Abstract

*Avicennia marina* is the most abundant and common mangrove species and has been used as a traditional medicine for skin diseases, rheumatism, ulcers, and smallpox. However, its anticancer activities and polyphenol contents remain poorly characterized. Thus, here we investigated anticancer activities of secondary *A. marina* metabolites that were purified by sequential soxhlet extraction in water, ethanol, methanol, and ethyl acetate (EtOAc). Experiments were performed in three human breast cancer cell lines (AU565, MDA-MB-231, and BT483), two human liver cancer cell lines (HepG2 and Huh7), and one normal cell line (NIH3T3). The chemotherapeutic potential of *A. marina* extracts was evaluated in a xenograft mouse model. The present data show that EtOAc extracts of *A. marina* leaves have the highest phenolic and flavonoid contents and anticancer activities and, following column chromatography, the EtOAc fractions F2-5, F3-2-9, and F3-2-10 showed higher cytotoxic effects than the other fractions. ^1^H-NMR and ^13^C-NMR profiles indicated that the F3-2-10 fraction contained avicennones D and E. EtOAc extracts of *A. marina* leaves also suppressed xenograft MDA-MB-231 tumor growth in nude mice, suggesting that EtOAc extracts of *A. marina* leaves may provide a useful treatment for breast cancer.

## INTRODUCTION

Cancer causes a wide variety of diseases and is characterized by uncontrolled growth and spread of abnormal cells and subsequent disruption of organ and tissue functions. Treatments for cancer usually include surgery followed by radiotherapies and chemotherapies [1] that inhibit cancer cell growth and proliferation. However, in contrast with localized surgery and radiotherapy, chemotherapy has systemic effects [2–4]. Among chemotherapeutic agents, phytochemicals, such as paclitaxel from the Pacific yew tree and vincristine from Vinca rosea Linn, are widely used to treat cancers and have demonstrated pro-apoptotic effects [5, 6]. Various chemopreventive and chemotherapeutic agents have been shown to induce apoptotic cell death in both *in vitro* and *in vivo* studies, suggesting that apoptosis plays a crucial role in cancer treatment [7]. Accordingly, the widely used chemotherapeutic drug 5-fluorouracil (5-FU) inhibits tumor cell growth in animal models by inducing apoptotic activation of the CD95/CD95L system [8]. The chemopreventive agent curcumin predominantly induces apoptosis via mitochondria-mediated pathways in various cancer cell types [9]. Apoptosis, or programmed cell death, is a physiological process that eliminates abnormal, misplaced, or nonfunctional cells and is critical for maintenance of tissue homeostasis [10]. Excessive apoptosis causes organ atrophy and dysfunction, whereas failure of apoptosis results in accumulation of abnormal cells, potentially leading to tumor development. Apoptosis is controlled at multiple molecular levels and involves pro- and anti-apoptotic members of the Bcl-2 protein family [11]. Many studies have shown that dietary phytochemicals induce apoptosis in cancer cells, suggesting potential for development as cancer therapeutic agents [12].

Mangrove forests are economically and ecologically important and are rich in medicinal and non-medicinal edible plants. In particular, mangroves produce a wide variety of structurally novel natural agents with biochemical profiles [13]. *Avicennia marina* is a mangrove species of the Acanthaceae family, and discoveries of its chemical compounds have received much attention [14]. *A. marina* has been used as a traditional medicine for the treatment of skin diseases, rheumatism, ulcers, and smallpox. *In vitro* antimalarial, antibacterial, analgesic, and cytotoxic activities of *A. marina* have been reported [15]. Hence, *A. marina* is considered a valuable source of chemical constituents with medicinal potential. Among these, luteolin 7-*O*-methylether, chrysoeriol 7-*O*-glucoside, isorhamnetin 3-*O*-rutinoside, tannin lapachol, and naphthoquinone analogs have been isolated, and their biological activities have been described [16]. However, few studies demonstrate *in vitro* and *in vitro* anti-cancer activities of *A. marina*, and its chemical constituents have not been extensively examined.

Here we evaluated the biomedical potential of *A. marina* plants from a mangrove forest and established the chemical composition of *A. marina* extracts. In particular, total phytopolyphenol contents were separated using chromatography, and *A. marina* fractions were evaluated for anti-cancer effects in *in vitro* and *in vivo* models.

## RESULTS

### Polyphenol contents and anticancer activities against breast and liver cancer cell lines

Plant extracts that are rich in polyphenols have been safely used as traditional Chinese medicines for many centuries. Thus, water (H_2_O), ethanol (EtOH), methanol (MeOH), and ethyl acetate (EtOAc) extracts of *A. marina* were evaluated for phenol and flavonoid contents. As shown in Table 1A, EtOAc extracts of *A. marina* leaves had the highest phenol (80.96 ± 0.78 mg/g) and flavonoid (18.6 ± 2.01 mg/g) contents, followed by H_2_O and MeOH extracts of *A. marina* leaves. Similarly, EtOAc extracts of *A. marina* seeds were richer in phenols and flavonoids than H_2_O and MeOH extracts. Although mineral contents in *A. marina* leaves have not been determined previously, these were similar to those reported in previous studies of medicinal plants. Specifically, inductively coupled plasma atomic emission spectroscopy (ICP-AES) revealed the presence of the trace metal elements lead (Pb), zinc (Zn), nickel (Ni), indium (In), iron (Fe), aluminum (Al), arsenic (As), copper (Cu), cadmium (Cd), chromium (Cr), and silver (Ag) in *A. marina* leaves that were collected from the Xinfeng mangrove conservation area in Taiwan. Average concentrations of Rb, Zn, Ni, In, Fe, and Al in *A. marina* leaves were 10.3, 15.5, 2.7, 2.6, 128.7, and 93.3 mg/kg, respectively (Table 1B). The absence of detectable As, Cu, Cd, Cr, and Ag in the present *A. marina* leaves was considered favorable for clinical application without toxicity.

**Table 1A:** Total phenol and flavonoid in *Avicennia marina* leaves extraction

A. marina Extracts							
Content of phytochemicals (mg/g)	Leaf				Seed		
	MeOH	EtOH	H_2_O	EtOAc	EtOH	H_2_O	EtOAc
**Total phenols**	46.96	22.82	47.06	80.96	49.96	36.08	82.23
	± 0.24	± 1.80	± 2.15	± 0.78	± 3.85	± 6.85	± 1.12
**Total flavonoids**	9.26	11.96	6.83	18.69	3.15	1.47	4.72
	± 1.05	± 3.16	± 1.57	± 2.01	± 1.02	± 0.08	± 0.58

**Table 1B:** Concentrations of trace metal elements in *Avicennia marina* leaves

	Ag	Al	As	Cu	Cd	Cr	Fe	Ni	In	Rb	Zn
	(mg/kg)^a^										
*A. marina*	ND^b^	93.3	ND	ND	ND	ND	128.7	2.7	2.6	10.3	12.7

a Concentration of trace metal elements (mg/kg dry matter) in *Avicennia marina* leaves.

b ND, not detected.

**Figure 1:**
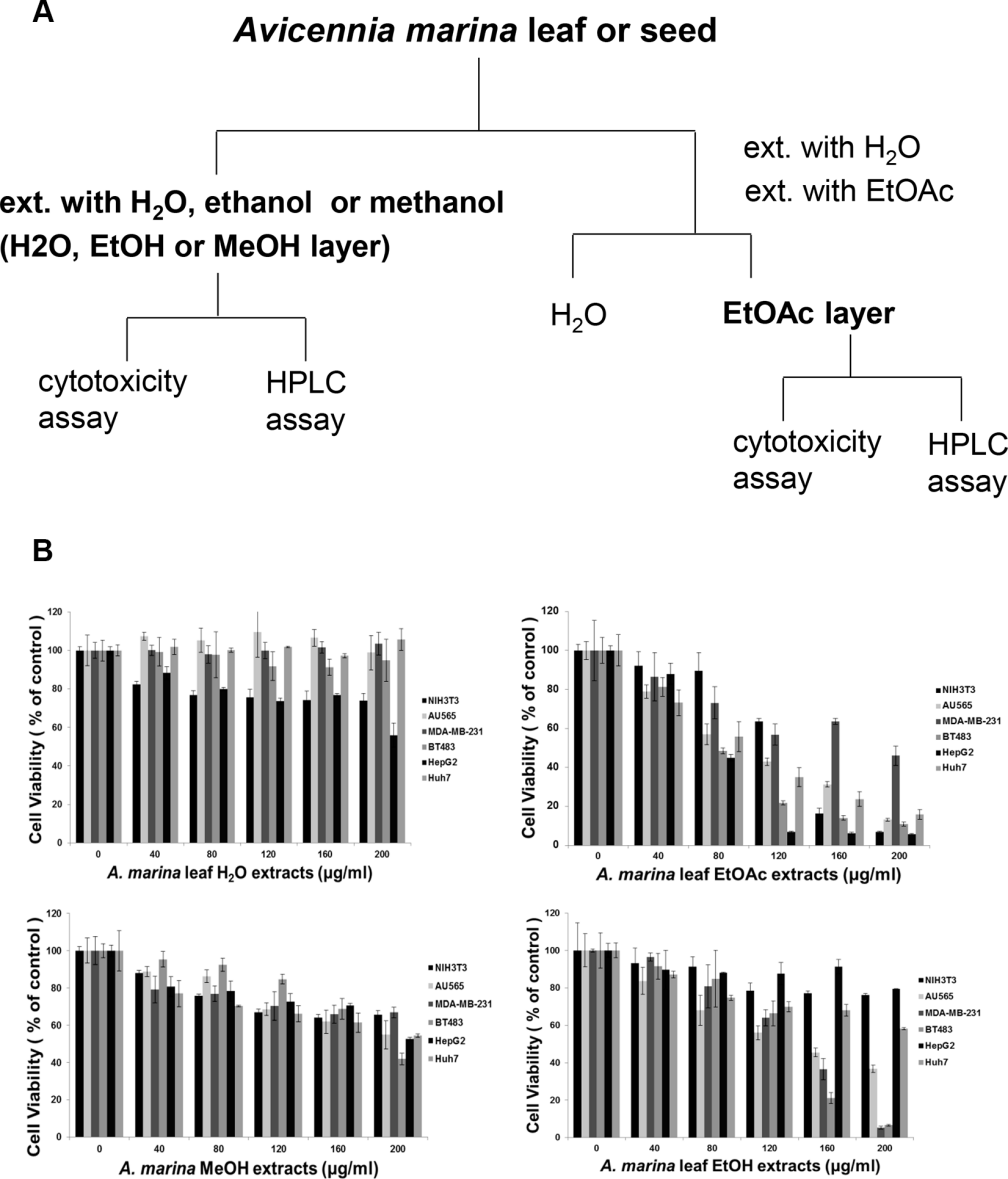
(**A**) Procedure for obtaining extracts from *Avicennia marina*; (**B**) Comparisons of cell viability after treatments with water (H_2_O), ethanol (EtOH), methanol (MeOH), and ethyl acetate (EtOAc) extracts of *A. marina* leaves; MTT assays showed differing responses of cell lines after 48-h treatments with differing extracts of *A. marina* leaves.

To determine whether high phenol and flavonoid contents were associated with anticancer activities, cytotoxic effects of H_2_O, EtOH, MeOH, and EtOAc extracts of *A. marina* leaves were compared using MTT assays in normal NIH3T3 cells, and in breast (AU565, MDA-MB-231 and BT483) and liver (HepG2 and Huh7) cancer cell lines (Figure 1B). In these experiments, EtOH and EtOAc extracts of *A. marina* leaves inhibited cell growth in cancer cell lines more than in normal NIH 3T3 cells. Inhibition of cancer cell growth was greater with EtOAc extracts than with EtOH and MeOH extracts, but was dose-dependent in all cases, and similar observations were made after treatments with EtOAc extracts of *A. marina* seeds (data not shown). However, whereas growth inhibition by H_2_O extracts of *A. marina* leaves was significant at 1000 μg/mL, H_2_O extracts had no effect at 200 μg/mL (data not shown). These data suggest that EtOAc extracts of *A. marina* leaves have the highest polyphenol contents and anticancer activities. Accordingly, EtOAc extracts did not inhibit proliferation of NIH 3T3 cells at 40–80-μg/mL, but significantly inhibited cell proliferation in AU565, BT483, HepG2, and Huh7 cancer cells. Thus, subsequent analyses were performed after treatments with EtOAc extracts at 40–80 μg/mL.

**Figure 2:**
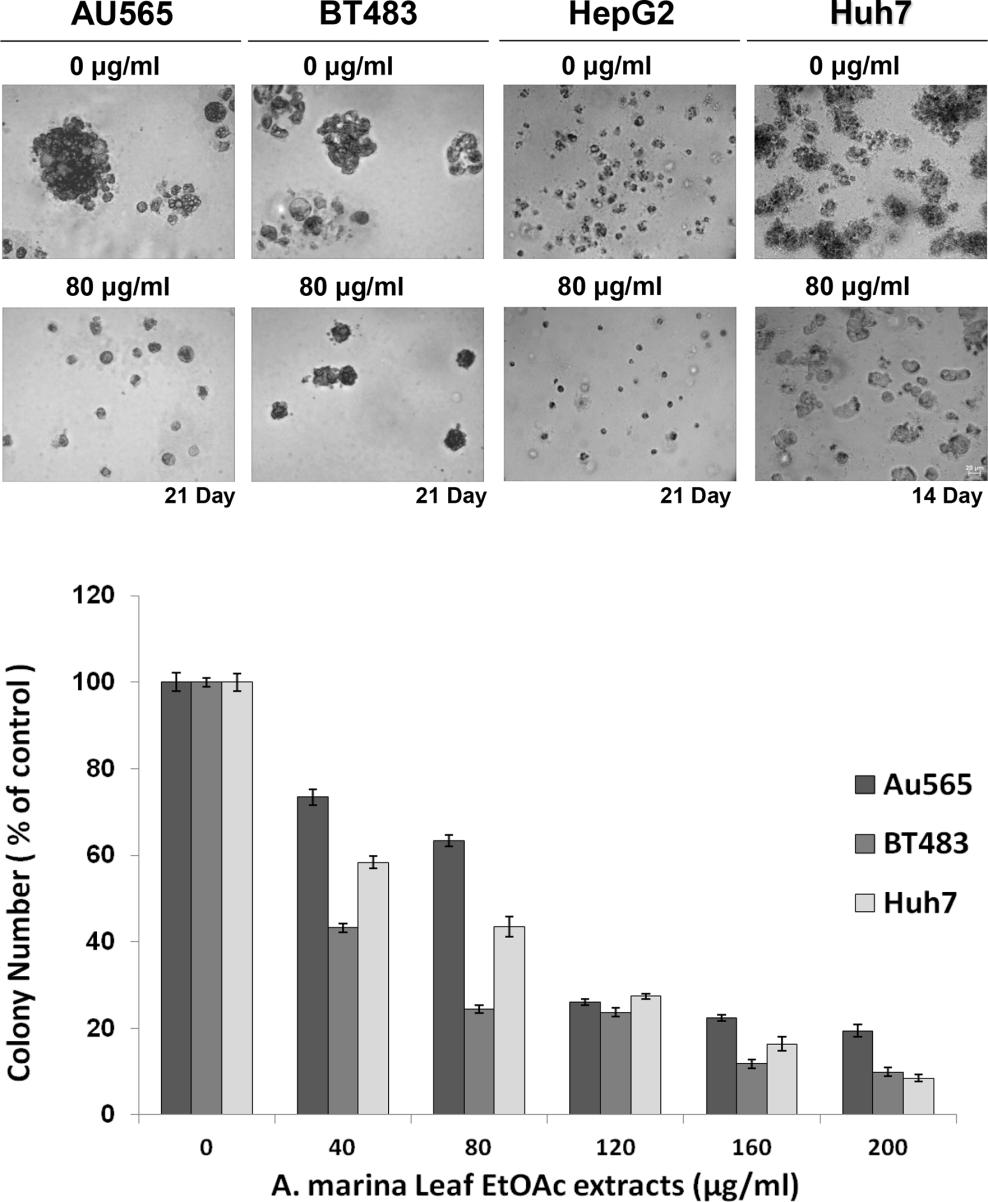
Effects of EtOAc extracts from *A. marina* leaves on anchorage-independent growth of AU565, BT483, HepG2, and Huh7 cancer cells Cells were treated with EtOAc extracts at 40–200 μg/mL in 0.35% agarose containing 10% FCS over 0.7% agarose containing 10% FCS. Cell colonies were observed after 14–21 days incubation at 37°C in 5% CO_2_ under light microscopy. Bars on images represent 20 m. Colonies of > 50 μm were counted after the incubation period and numbers are presented as percentages of the control.

Soft agar colony formation assays can be used to separate cancer cells and to confirm phenotypes, and were used to determine the effects of EtOAc extracts of *A. marina* leaves on anchorage-independent growth in breast (AU565 and BT483) and liver (HepG2 and Huh7) cancer cells. After 14–21 days of treatment with the EtOAc extract, cultured cells showed reduced soft agar colony formation, with decreased colony sizes and a smaller numbers of AU565, BT483, HepG2, and Huh7 cancer cell colonies (Figure 2).

### Chemical composition of EtOAc extracts of *A. marina* leaves

**Figure 3:**
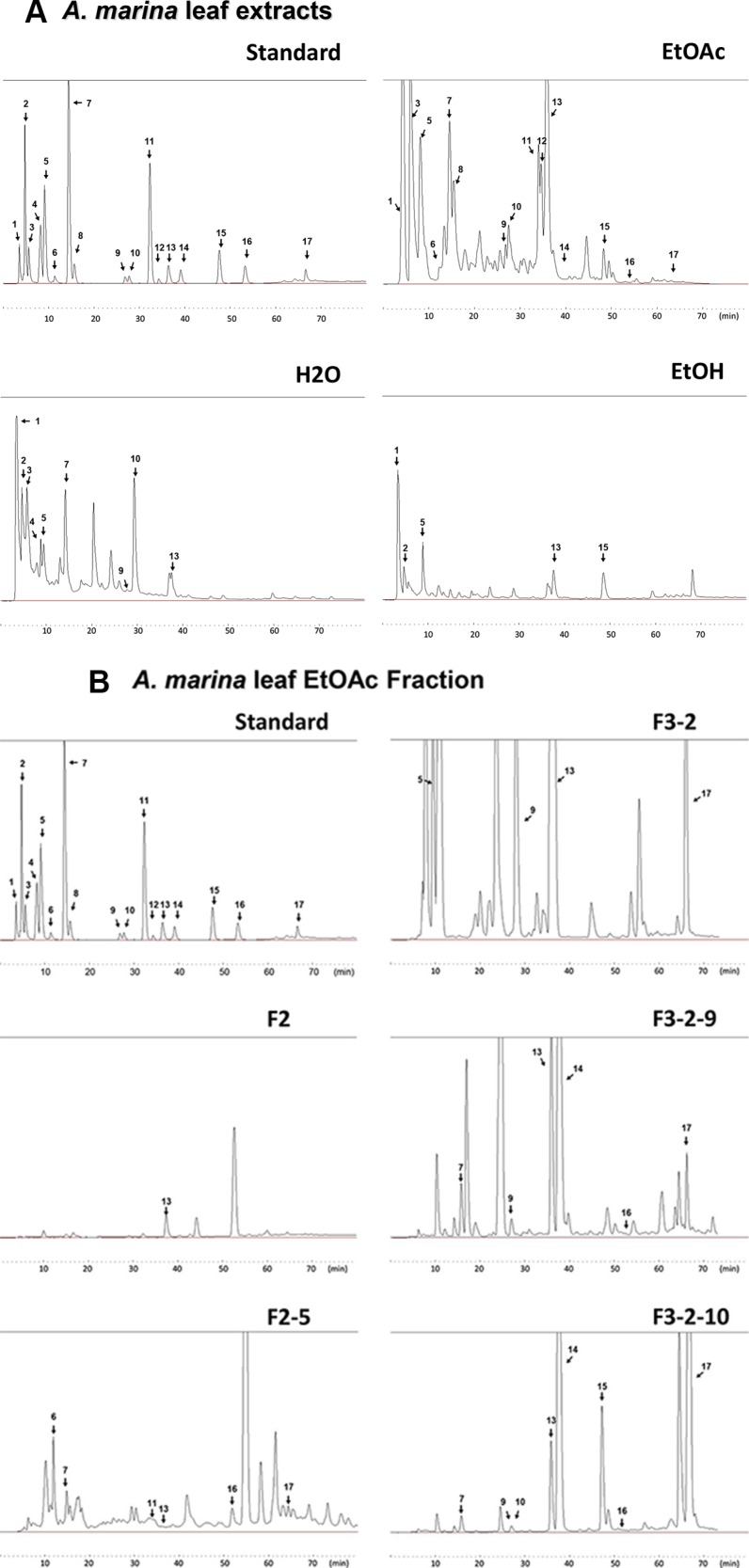
Analyses of polyphenol contents in *A. marina leaves* (**A**) Polyphenol contents of H_2_O, EtOAc, and EtOH extracts of *A. marina* leaves were analyzed using HPLC; HPLC chromatograms of the polyphenol standard mixture were recorded at 280 nm; Peaks: 1, ellagic acid; 2, ellagic acid + gallic acid; 3, chlorogenic acid; 4, catechin; 5, syringic acid + epigallocatechin gallate (EGCG) + rutin + caffeic acid; 6, corilagin; 7, narigenin + p-coumaric acid; 8, picatechin gallate (ECG); 9, myricetin; 10, theaflavin (TF1); 11, resveratrol; 12, theaflavin-3-gallate (TF2); 13, luteolin; 14, quercetin; 15, apigenin; 16, kaempferol; 17, chrysin. (**B**) Chromatograms of fractions separated from EtOAc extracts of *A. marina* leaves.

Polyphenol concentrations of H_2_O, EtOH, and EtOAc extracts of *A. marina* leaves were determined using HPLC, and included the 14 flavonoids apigenin, chrysin, catechin, epigallocatechin gallate (EGCG), picatechin gallate (ECG), kaempferol, luteolin, narigenin, myricetin, quercetin, rutin, resveratrol, theaflavin (TF1), and theaflavin-3-gallate (TF2), and the 7 phenolic acids caffeic acid, chlorogenic acid, p-coumaric acid, corilagin, ellagic acid, gallic acid, and syringic acid (Figure 3A and Table 2A).

**Table 2A:** Contents (micrograms per gram) of phenolic acids in different extracts of *A. marina* leaf

Peak	Retention time (min)	Leaf (EtOAc)	Leaf (H_2_O)	Leaf (EtOH)	Comparison with standard
**1.**	3.52	40.367 ± 1.232	28.102 ± 1.369	19.331 ± 2.133	Elliagic Acid (1)
**2.**	4.72	ND	1.984 ± 0.228	0.276 ± 0.013	Elliagic Acid (2), Gallic Acid
**3.**	5.55	38.60 ± 2.117	8.918 ± 0.139	ND	Chlorogenic Aicd
**4.**	8.17	ND	2.149 ± 1.131	ND	Catechin
**5.**	9.03	31.120 ± 1.01	7.011 ± 1.237	6.051 ± 2.526	Syring Acid, EGCG, Rutin, Caffeic Acid
**6.**	11.28	2.123 ± 1.231	ND	ND	Corilagin
**7.**	14.35	10.214 ± 0.912	5.854 ± 0.243	ND	Naringic, p-Coumaric Acid
**8.**	15.60	40.062 ± 2.117	ND	ND	ECG
**9.**	26.78	11.183 ± 0.182	9.985 + 2.496	ND	Myricetin
**10.**	27.67	19.303 ± 2.362	39.837 ± 5.199	ND	TF1
**11.**	32.25	6.487 ± 0.325	ND	ND	Resveratrol
**12.**	34.22	156.381 ± 5.475	ND	ND	TF2
**13.**	36.32	29.335 ± 2.311	2.298 ± 0.011	4.504 + 1.715	Luteolin
**14.**	39.03	0.941 ± 0.140	ND	ND	Quercetin
**15.**	47.55	4.924 ± 0.987	ND	3.82 ± 0.615	Apigenin
**16.**	53.22	0.231 ± 0.009	ND	ND	Kaempferol
**17.**	66.55	0.810 ± 0.012	ND	ND	Chrysin

### Identification and separation of EtOAc extracts of *A. marina* leaves

**Figure 4:**
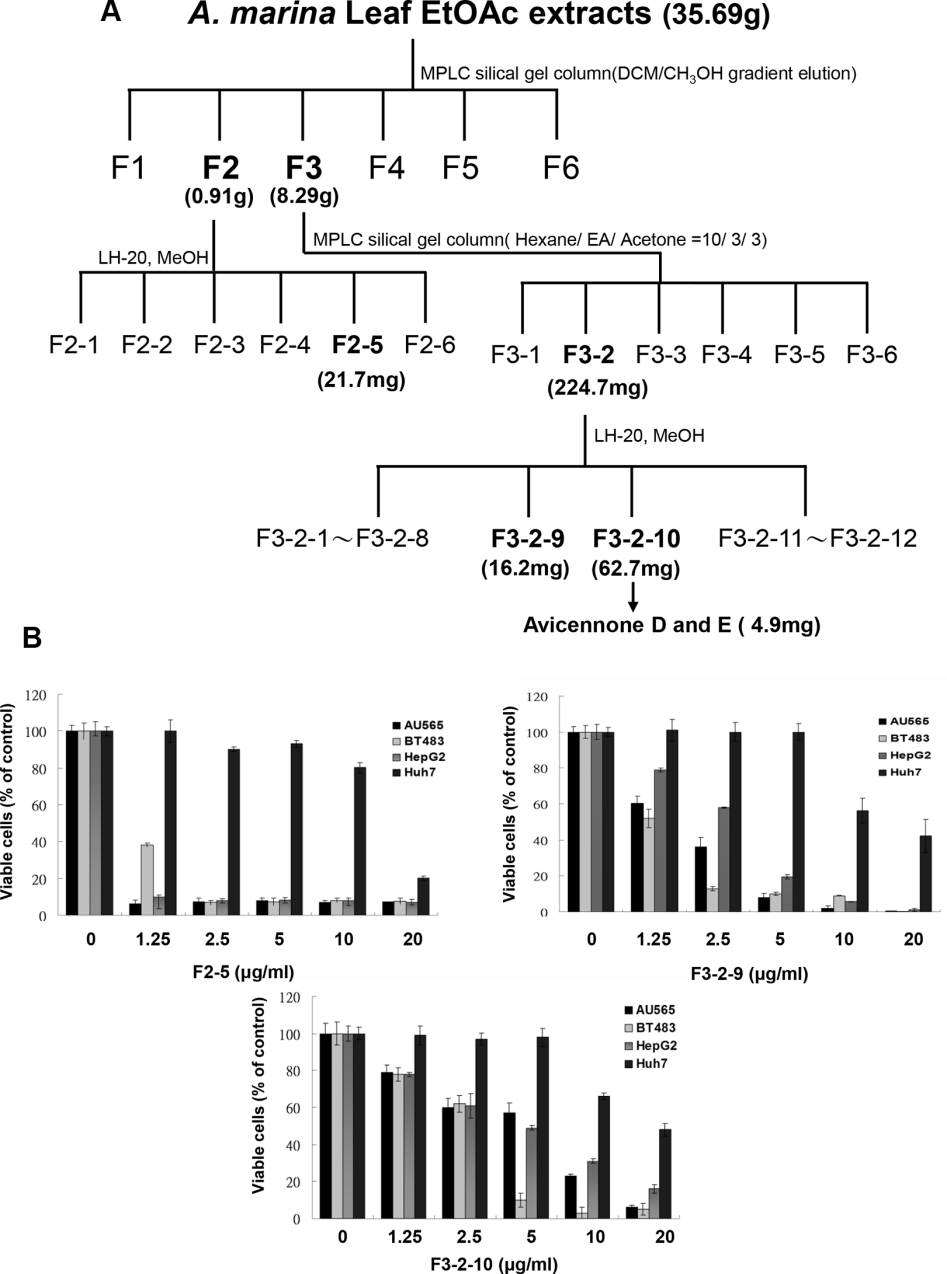

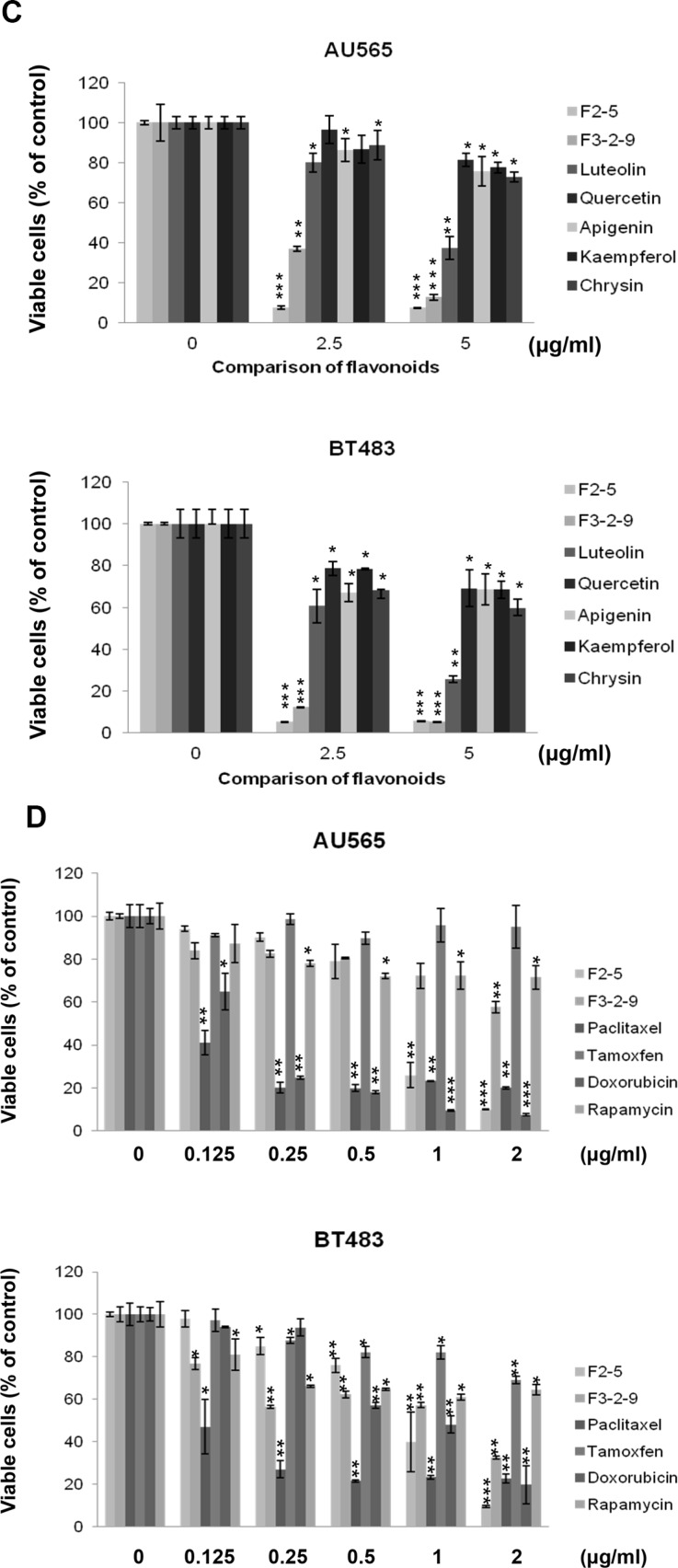
(**A**) Procedure for fractionation of EtOAc extracts from *A. marina* leaves; (**B**) Antiproliferative effects of F2-5, F3-2-9, and F3-2-10 in AU565, BT483, HepG2, and Huh7 cancer cells were determined using MTT assays. (**C**) Comparisons of antiproliferative effects of F2-5, F3-2-9, luteolin, quercetin, apigenin, kaempferol, and chrysin in AU565 and BT483 breast cancer cells; (**D**) Comparisons of antiproliferative effects of F2-5, F3-2-9, paclitaxel, tamoxifen, tdoxorubicin, and rapamycin on AU565 and BT483 breast cancer cells. All values are expressed as means ± standard errors of the mean (SE) and differences were identified using *t*-tests; *p < 0.05, **p < 0.01, ***p < 0.001.

To identify bioactive components of *A. marina* leaves, EtOAc extracts were purified using column chromatography (Figure 4A). The plant material (20 kg) of *A. marina* was immersed in 200 L distilled water for 30 min and then boiled at 95°C for 30 min. The dried extract was partitioned between H_2_O and EtOAc to yield 35.69 g of a dried EtOAc extract and an aqueous residue. The EtOAc extract was subjected to silica gel medium pressure column chromatography (gradient mixtures, from 100% CH_2_Cl_2_ to 100% MeOH). The eluents were pooled to form 6 fractions on the basis of TLC analysis. Fraction 3 (8.29 g) was further purified on a silica gel column chromatography (hexane-EtOAc-Acetone, 10:3:3) to give fractions F3-1 to F3-6. Fraction F3-2 (224.7 mg) was subjected to Sephadex LH-20 column chromatography (MeOH) to give fractions F3-2-1 to F3-2-12.

Results showed that the fractions 2-5 (F2-5), 3-2-9 (F3-2-9), and 3-2-10 (F3-2-10) from EtOAc extracts of *A. marina* leaves had the highest anticancer effects (Figure 4B). Specifically, percentage inhibition was observed in the order F2-5 > F3-2-9 > F3-2-10, and F2-5 significantly inhibited cell proliferation in AU565, BT483, HepG2, and Huh7 cells in a dose-dependent manner, with half inhibitory concentrations (IC_50_) of 0.75, 0.85, 0.79, and 15.6 μg/mL, respectively. Similar results were observed following treatments with F3-2-9 and F3-2-10, with IC_50_ values of 2.1, 1.2, 3.2, and 9.8 μg/mL, and 5.6, 3.8, 5.1, and 19.2 μg/mL, respectively. In further HPLC analyses of F2-5, F3-2-9, and F3-2-10 (Figure 3B and Table 2B), F3-2-9 contained two major HPLC peaks with retention times of 36–40 min that corresponded with luteolin and quercetin standards. The major HPLC peak from F2-5 had a retention time of 55 minutes, and did not match any of the 21 standards. Subsequent NMR analyses showed that the most abundant components were the same in F3-2-9 and F3-2-10, but differed from those in F2-5 (data not shown).

**Table 2B:** Contents (micrograms per gram) of phenolic acids in EtOAc extracts fraction of *A. marina* leaf

Peak	Retention time (min)	F2	F2-5	F3	F3-2-9	F3-2-10	Comparison with standard
**1.**	3.52	ND	ND	ND	ND	ND	Elliagic Acid (1)
**2.**	4.72	ND	ND	ND	ND	ND	Eliiagic Acid (2), Gallic Acid
**3.**	5.55	ND	ND	ND	ND	ND	Chlorogenic Aicd
**4.**	8.17	ND	ND	ND	ND	ND	Catechin
**5.**	9.03	ND	ND	38.720	ND	ND	byring Acia, LUUU, Kutin, Caffeic Acid
**6.**	11.28	ND	237.840	ND	ND	ND	Corilagin
**7.**	14.35	ND	6.845	ND	0.234	0.367	Naringic, p-Coumaric Acid
**8.**	15.60	ND	ND	ND	ND	ND	ECG
**9.**	26.78	ND	ND	686.320	3.890	2.361	Myricetin
**10.**	27.67	ND	ND	ND	ND	0.697	TF1
**11.**	32.25	ND	0.123	ND	ND	ND	Resveratrol
**12.**	34.22	ND	ND	ND	ND	ND	TF2
**13.**	36.32	2.234	0.332	92.130	38.110	19.653	Luteolin
**14.**	39.03	ND	ND	ND	98.321	88.652	Quercetin
**15.**	47.55	ND	ND	ND	ND	38.146	Apigenin
**16.**	53.22	ND	1.328	ND	0.653	0.563	Kaempferol
**17.**	66.55	ND	1.398	110.690	22.095	132.780	Chrysin

The bioactive fraction F3-2-10 further purified by HPLC to yield 1 and 2 (4.9 mg). The chemical structures of compounds 1 and 2 were identified by NMR spectroscopy. Compounds 1 and 2, which were chromatographically inseparable could be obtained only as a mixture (1:1, according to ^1^H & ^13^C NMR data). The identical molecular formula of 1 and 2 was established as C_12_H_6_O_4_ by ESIMS at m/z 213.0 [M-H]^−^ and NMR data. A naphthoquinone skeleton was suggested by the absorption bands of UV spectrum at λ_max_ 267, 307, and 350 nm. One set of ^1^H NMR signals of compound 1 indicated three aromatic protons (δ_H_ 8.01, 7.47, 7.10) in an AMX spin system and two AB-type olefinic protons (δ_H_ 7.96, d, J = 1.9 Hz, δ_H_ 6.99, d, J = 1.9 Hz) of a furan ring (Table 3). In the ^13^C NMR spectrum, two sets of signals, each representing 12 carbons, could be distinguished. The NMR data of the mixture of compounds 1 and 2 indicated them to carry a hydroxyl substituent on the aromatic ring. Based on above data and with those reported in the literature, 1 was determined to be 6-hydroxynaphtho [2, 3-*b*] furan-4, 9-dione and 2 as 7-hydroxynaphtho [2, 3-*b*] furan-4, 9-dione, and these compounds were named avicennone D and avicennone E, respectively. In contrast with previously reported ^1^H-NMR and ^13^C-NMR data, the F3-2-10 fraction was identified as a mixture of avicennone D and E (Table 3). To our knowledge, this is the first study to demonstrate apoptotic mechanisms of avicennone D and E from a HPLC fraction from an EtAOc extract of *A. marina* leaves in breast and liver cancer cells.

**Table 3:** ^1^H NMR and ^13^C NMR data of avicennone D (1) and avicennone E (2) (CD_3_OD)^a^

atom number	1		2	
	δ_C_ (mult.)	δ_H_ (mult. *J* in Hz)	δ_C_ (mult.)	δ_H_ (mult. *J* in Hz)
2	150.29 (d)	7.96 (d, 1.9)	150.82 (d)	7.98 (d, 1.9)
3	109.17 (d)	6.99 (d, 1.9)	109.40 (d)	6.99 (d, 1.9)
3a	131.32 (s)		132.07 (d)	
4	181.93 (s)		181.24 (t)	
4a	137.12 (s)		126.56 (s)	
5	114.44 (d)	7.47 (d, 2.6)	130.68 (d)	8.03 (d, 8.5)
6	164.73 (s)		121.10 (d)	7.12 (dd, 8.5, 2.6)
7	121.2 (d)	7.10 (dd, 8.5, 2.6)	164.78 (s)	
8	130.55 (d)	8.01 (d, 8.5)	114.14 (d)	7.49 (d, 2.6)
8a	125.73 (s)		136.29 (s)	
9	174.42 (s)		174.81 (s)	
9a	154.56 (s)		154.06 (s)	

a Reference to residual solvent CD_3_OD signals at δ_H_ 3.3 and δ_C_ 49.0 and measured at 25°C, 600 MHz for ^1^H and 150 MHz for ^13^C. ^13^C multiplicities were assigned from DEPT experiments.


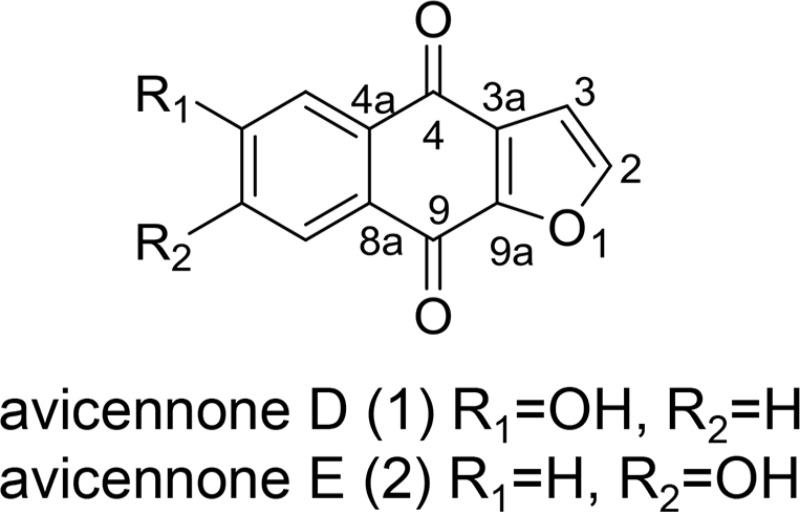


Major HPLC peaks from F2-5, F3-2-9, and F3-2-10 showed retention times of 36–66 minutes, and corresponded with luteolin, quercetin apigenin, kaempferol, and chrysin standards (Figure 3B and Table 2B). Thus, the anticancer properties of these polyphenols were compared with those of F2-5 and F3-2-9 in AU565 and BT483 cells. In these experiments, anticancer activities were ranked in the same decreasing order of F2-5 > F3-2-9 > luteolin > quercetin, apigenin, kaempferol, and chrysin in both cell lines (Figure 4C). In comparisons with paclitaxel, tamoxifen, doxorubicin, and rapamycin, antiproliferative activities decreased in the order paclitaxel > doxorubicin > F2-5 > F3-2-9 or rapamycin > tamoxifen in both cell lines. Similar results were also observed in BT483 cells (Figure 4D). Taken together, these data indicate the efficacy of F2-5 and F3-2-9 as treatments for breast cancer.

### Fractions of EtOAc extracts from *A. marina* leaves induced apoptosis in breast and liver cancer cell lines

**Figure 5:**
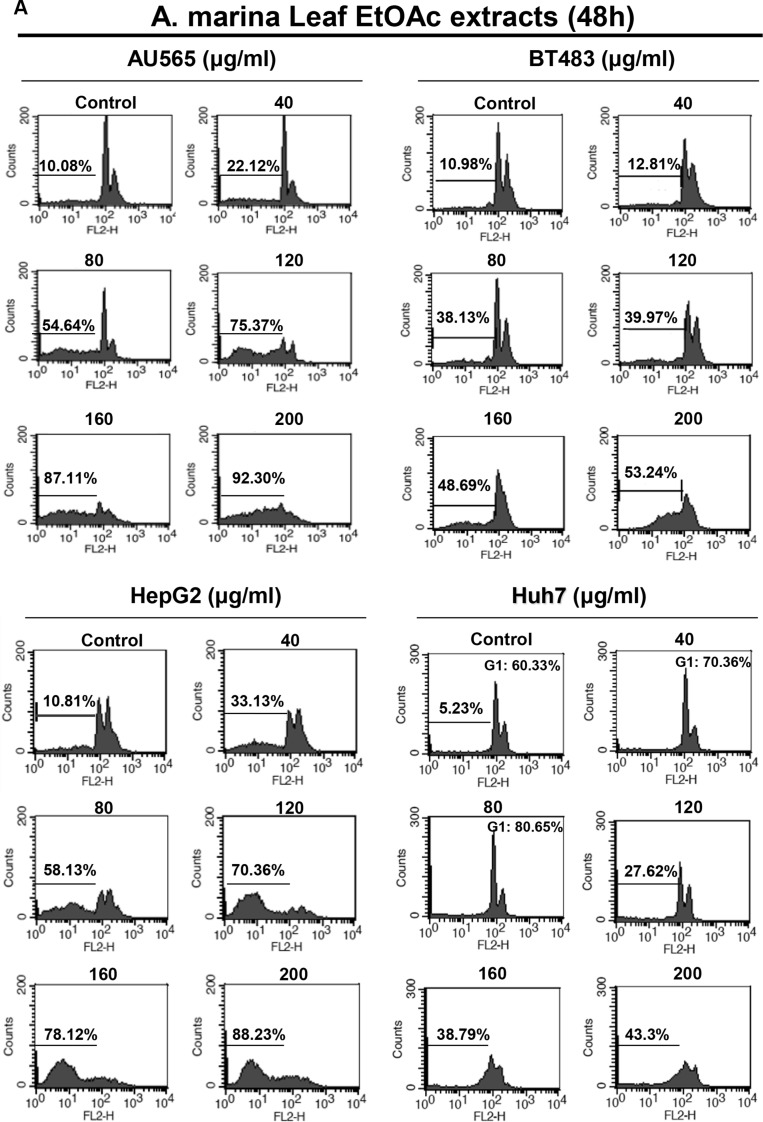

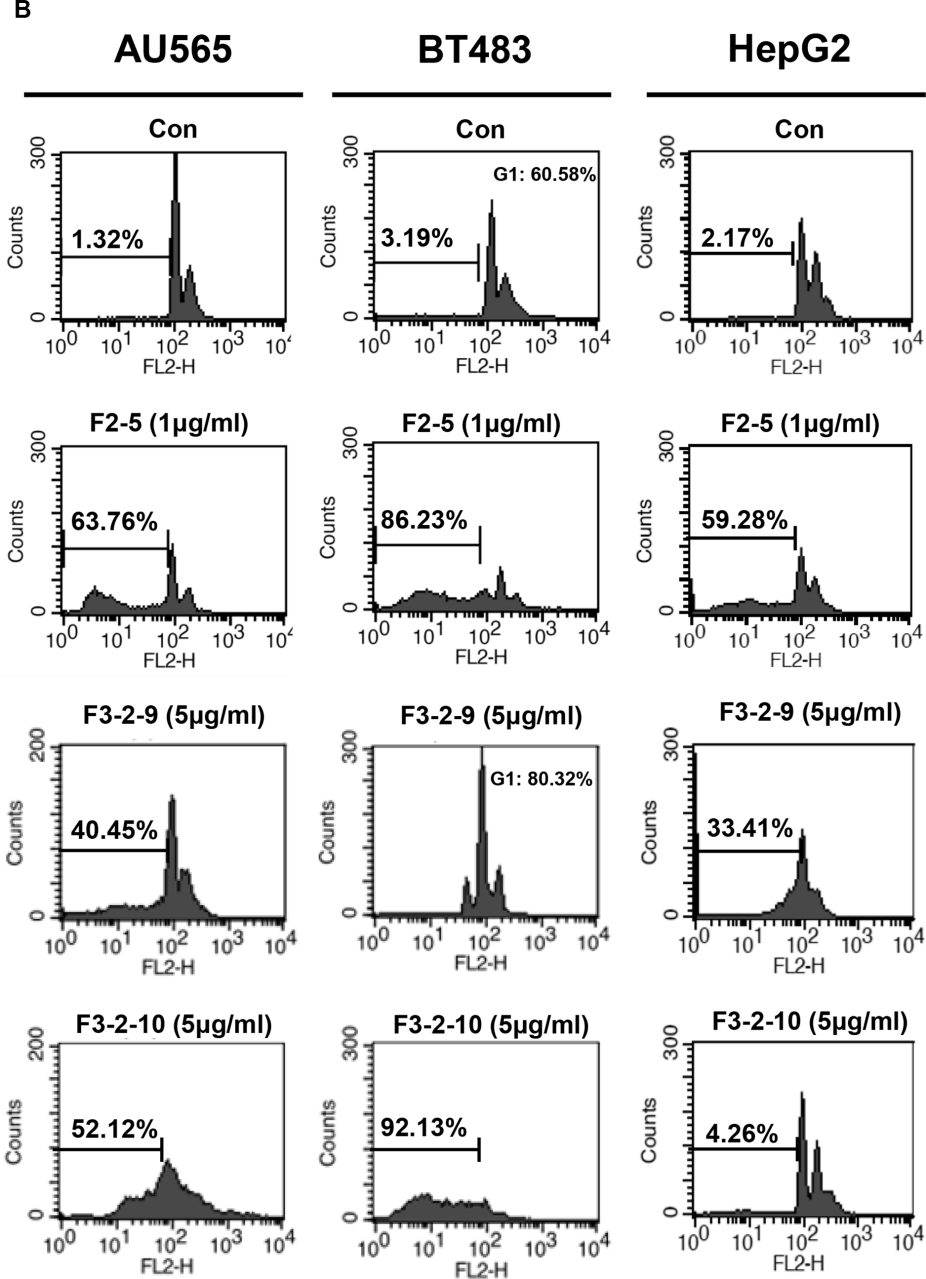
Effects of (A) EtOAc extracts of *A. marina* leaves and (B) fractions F2-5, F3-2-9, and F3-2-10 on cell cycle distributions of AU565, BT483, HepG2, and Huh7 cancer cells Cell cycle distributions were assessed using flow cytometry.

**Figure 6:**
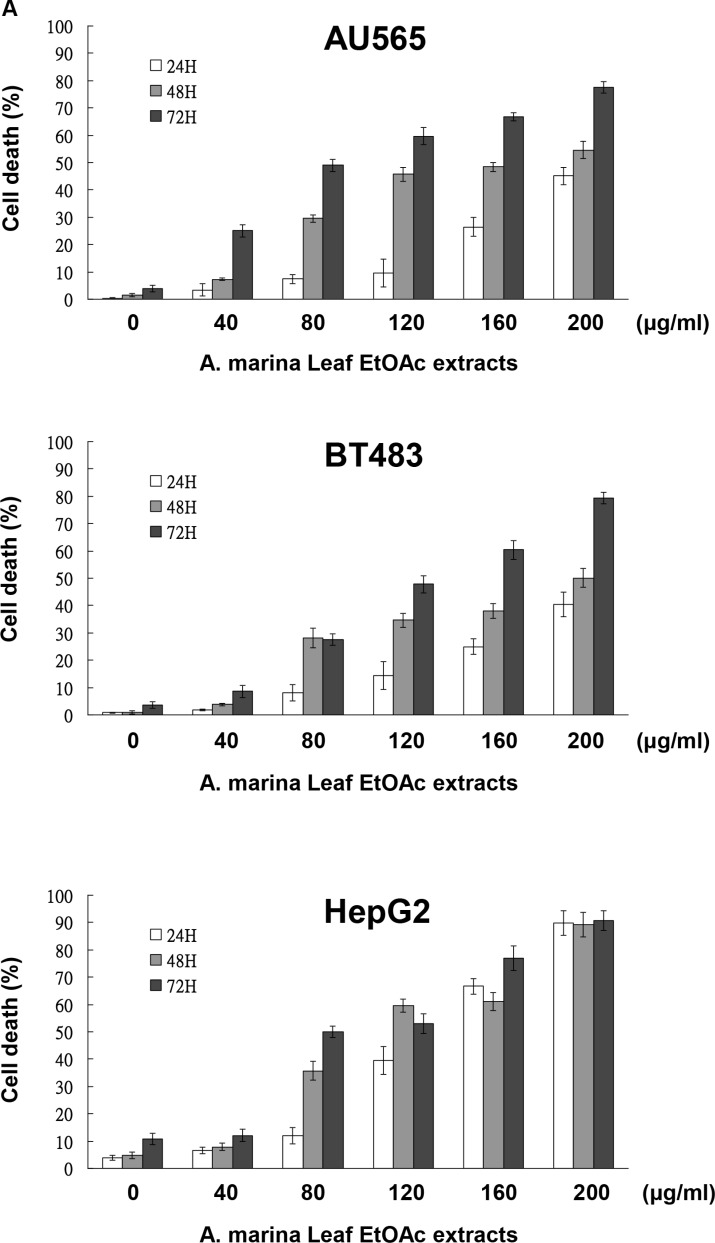

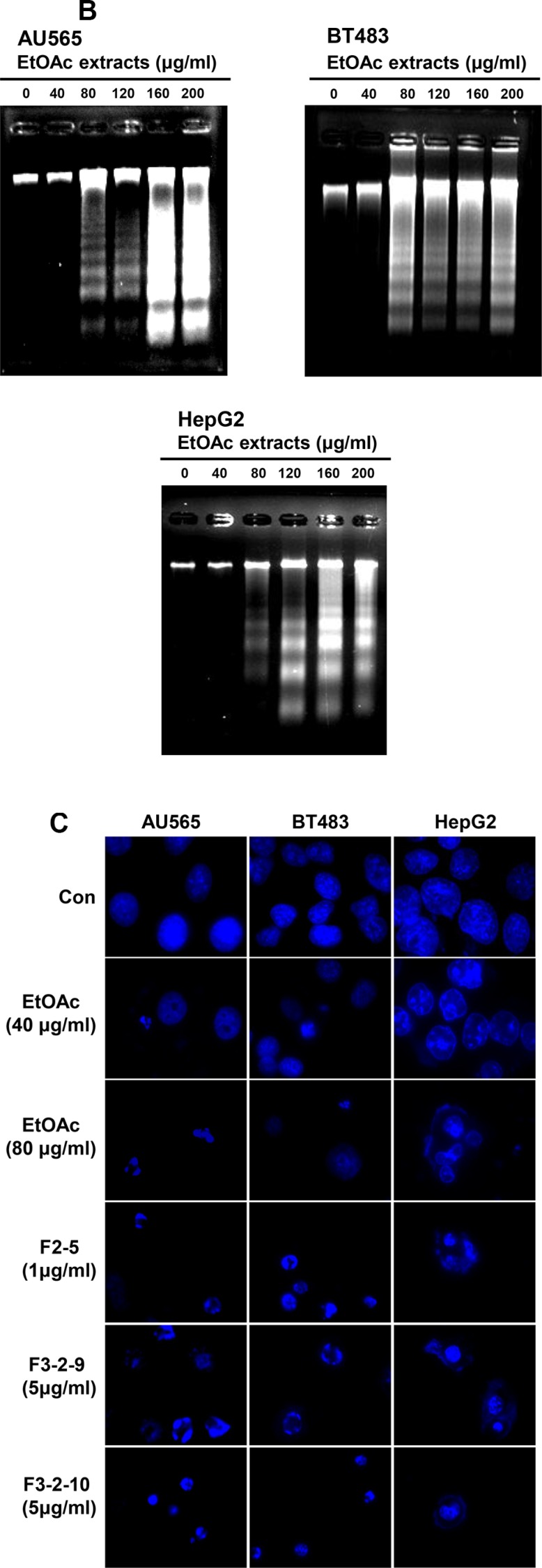

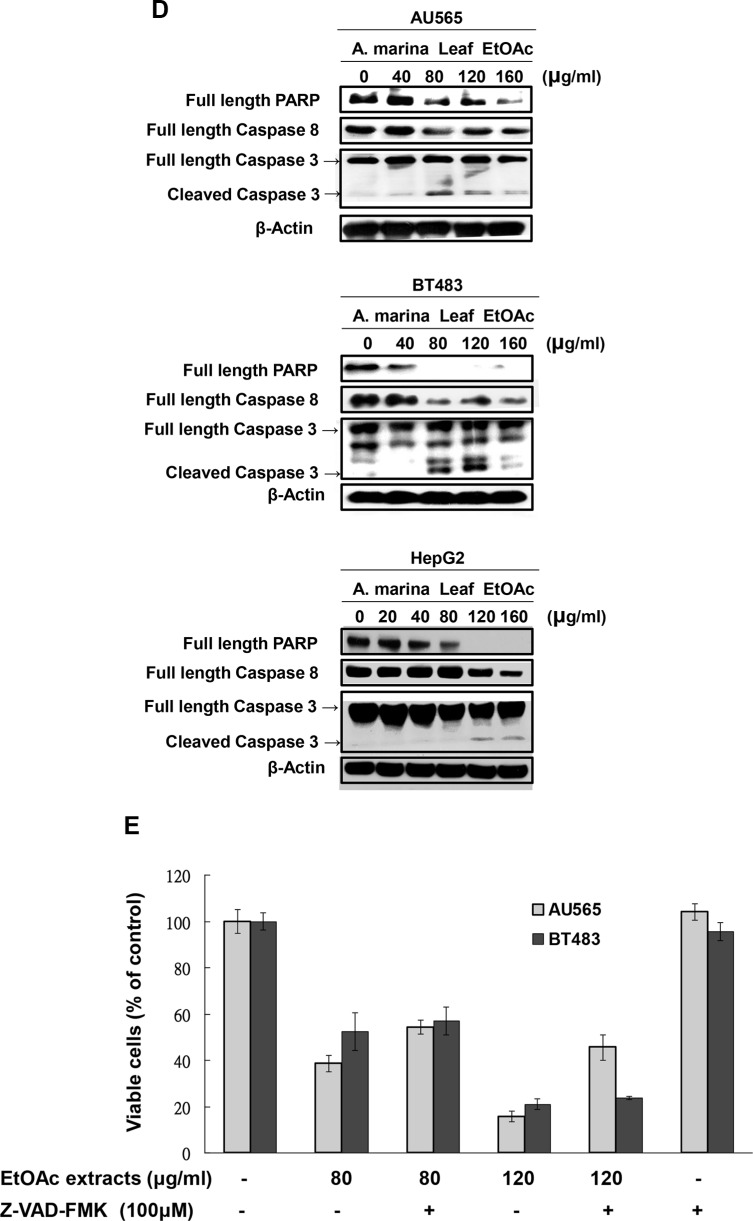
EtOAc extracts of *A. marina* leaves induced apoptosis in AU565, BT483, and HepG2 cells Cytotoxic effects of EtOAc extracts of *A. marina* leaves in AU565, BT483, and HepG2 cancer cells were determined using trypan blue dye exclusion assays (**A**), DNA fragmentation assays (**B**), and Hoechst 33258 Dye assays (**C**) as described in the Materials and Methods. (**D**) Effects of EtOAc extracts of *A. marina* leaves on full-length PARP, full-length caspase 8, and cleaved caspase 3 production in AU565, BT483, and HepG2 cancer cells; Cells were treated with various concentrations of EtOAc extracts for 48 h in 2% FBS media. Total protein was collected and western blotting analyses were performed as described in the Materials and Methods. (**E**) Effects of EtOAc extracts of *A. marina* leaves on AU565 and BT483 cancer cell viability with or without pretreatment with Z-VAD-FMK caspase inhibitor. Cell viability was determined using MTT assays.

To identify antiproliferative mechanisms of EtOAc extracts and their fractions, cell cycle analyses were performed in breast and liver cells after 48-h treatments using flow cytometry. Treatment of AU565, BT483, and HepG2 cells with 80–200-μg/mL EtOAc extracts for 48 h significantly increased percentages of cells in the sub-G1 phase (44.56%–82.22%; Figure 5A). However, Huh7 cells treated with EtOAc extracts for 48 h were arrested in the G1 phase at lower doses (40–80 μg/mL), and apoptosis was observed after treatments with 120–200-μg/mL EtOAc extract, and after treatments with 1-μg/mL F2-5, 5-μg/mL F3-2-9, or 5-μg/mL F3-2-10 (Figure 5B). In further experiments, apoptosis was assessed in AU565, BT483, and HepG2 cells after treatments with EtOAc extracts (40–200 μg/mL) using trypan blue exclusion, Hoechst, and DNA fragmentation assays. In these experiments, significant time- and dose-dependent cell death was observed in trypan blue exclusion assays (Figure 6A) and corresponded with dose-dependent DNA fragmentation (Figure 6B). Fluorescence photomicrographs of cells stained with Hoechst 33258 confirmed the induction of apoptosis following treatment with EtOAc extracts at 40–80 μg/mL for 48 h. Specifically, control cells showed round and homogeneous nuclei, whereas cells treated with 80-μg/mL EtOAc extracts showed condensed and fragmented nuclei (Figure 6C). Similar observations were made in cells treated with 1-μg/mL F2-5, 5-μg/mL F3-2-9, or 5-μg/mL F3-2-10.

Flow cytometry and DNA fragmentation data indicated that treatments with EtOAc extracts trigger apoptosis in AU565, BT483, and HepG2 cells. Accordingly, western blot analyses showed decreased protein expression of full-length PARP, full-length caspase 8, and increased expression of cleaved caspase 3 after treatment with 80-μg/mL EtOAc extracts for 48 h (Figure 6D). In further investigations of caspase-dependent mechanisms (Figure 6E), pretreatment of AU565 cells with the caspase inhibitor Z-VAD-FMK blocked EtOAc extract-mediated apoptosis, but did not block apoptosis in BT483 cells. These results suggest roles of both caspase-dependent and -independent mechanisms in BT483 cells.

### Anti-motility activities of EtOAc extracts of *A. marina* leaves in MDA-MB-231 cells

**Figure 7:**
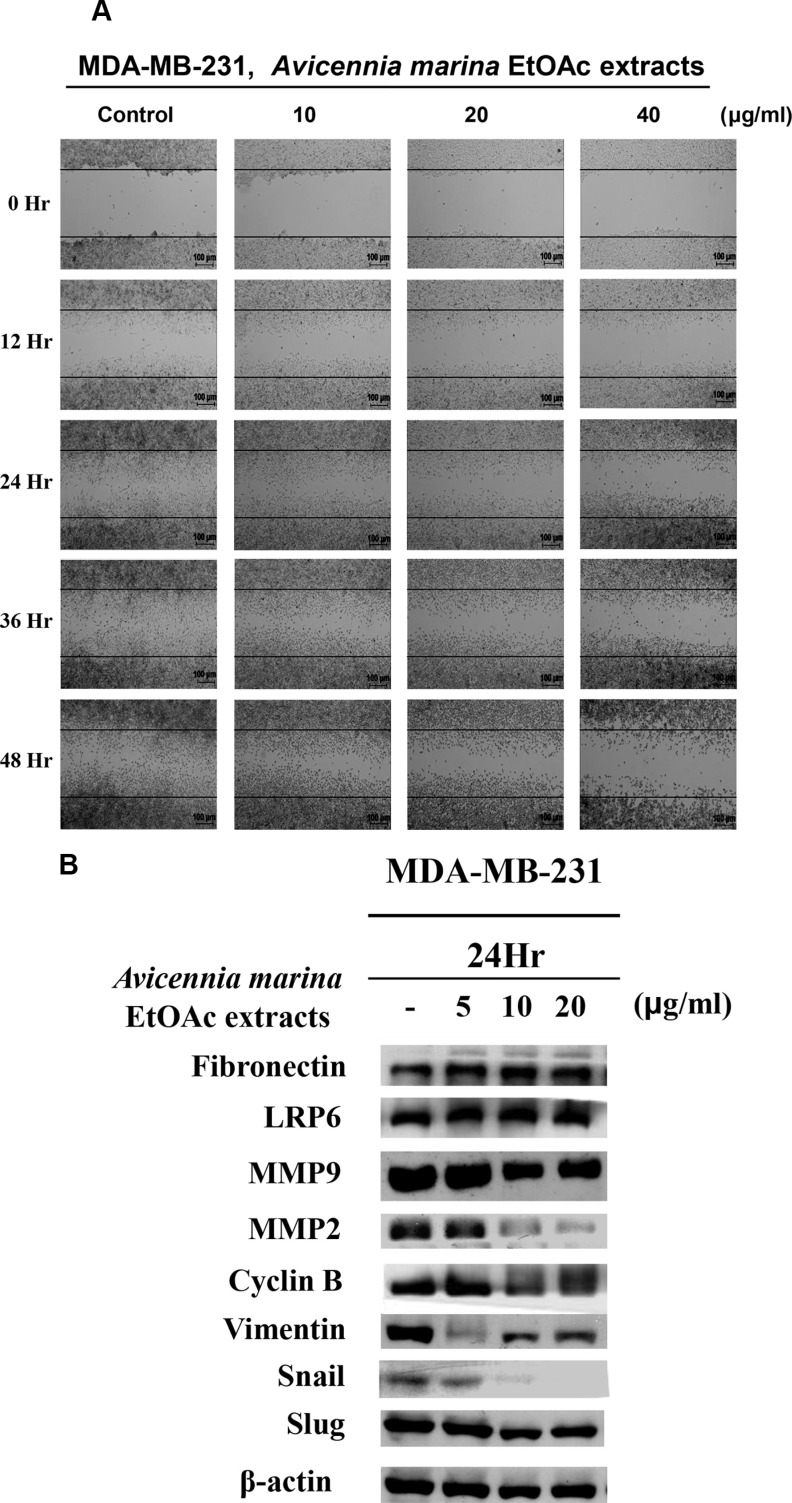
(**A**) Effects of 0–48-h treatments with EtOAc extracts of *A. marina* leaves on migration of MDA-MB-231 cells were examined using wound-healing assays. (**B**) LRP6, MMP9, MMP2, cyclin B, vimentin, snail protein, slug protein expression was determined using western blotting after 24-h exposures to various concentrations of EtOAc extracts of *A. marina* leaves as indicated.

The effects of EtOAc extracts of *A. marina* leaves on cell motility were examined using wound-healing assays. In these experiments, significant cytotoxic activity was observed in MDA-MB-231 cells after treatment with 80-μg/mL EtOAc extracts for 48 h. To decrease interference in assessments of motility, wound-healing assays were performed after 24–48 h exposure to 10–40 μg/mL EtOAc extracts (Figure 7A). These assays showed inhibition of motility but not growth in MDA-MB-231 cells. Because cell motility has been associated with expression of specific cell adhesion proteins such as MMP2, MMP9, and EMT-related proteins, expression levels of these and fibronectin, snail protein, slug protein, vimentin, LRP6, and cyclin B were determined in MDA-MB-231 cells. In these experiments, treatments with 10–20-μg/mL EtOAc extracts decreased MMP2, MMP9, cyclin B, vimentin, and snail protein expression in MDA-MB-231 cells after 24 h (Figure 7B).

### EtOAc and EtOH extracts, and F2-5 from *A. marina* leaves inhibited growth of MDAMB-231 xenograft tumors in nude mice

**Figure 8:**
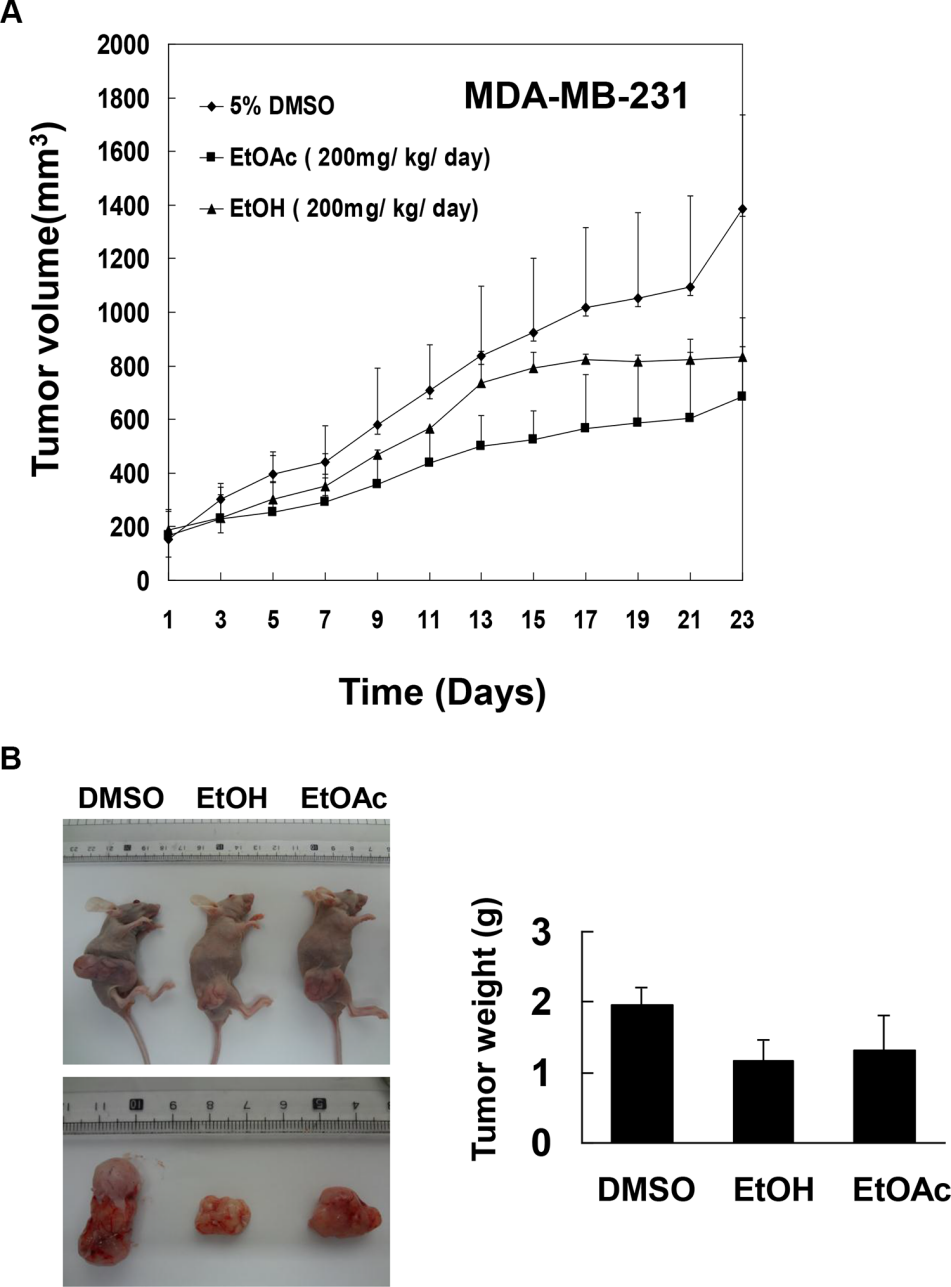
Effects of EtOAc and EtOH extracts of *A. marina* leaves on tumor growth in nude mice carrying MDAMB-231 xenografts Cells were subcutaneously implanted into the legs of female nude mice to induce tumor xenografts. Treatment groups were intraperitoneally injected with dimethyl sulfoxide (DMSO), EtOAc extracts (200 mg/kg/day), or EtOH extracts (200 mg/kg/day) for 23 days, and tumor volumes (**A**) and tumor weights (**B**) were determined.

To determine whether *A. marina* extracts inhibit breast cancer cell growth *in vivo*, human MDAMB-231 breast cancer cells were xeno-transplanted into the back skin of BALB/c nude mice and the effects of EtOAc extracts and EtOH extracts of *A. marina* leaves were investigated (Figure 8). In these animals, mean tumor volumes and weights were decreased by treatments with EtOAc and EtOH extracts at 200 mg/kg/day compared with DMSO-treated controls on day 23. However, no significant changes in relative weights of hearts, livers, spleens, or kidneys were observed in comparisons of EtOAc and EtOH extract-treated animals and the DMSO-treated control group. Finally, no differences in serum aspartate aminotransferase (AST) and creatinine values were found between treatment and control groups (data not shown), indicating limited side effects of *A. marina* extracts.

## DISCUSSION

Mangroves grow under environmental conditions of high visible and UV radiation [17]. Both visible and UV radiation have been shown to be harmful to the photosynthetic apparatus in plants. Accordingly, higher plants adapt to high irradiance by accumulating UV-B-absorbing phenolic compounds such as flavonoids. Such flavonoids and phenolic acids also have antioxidant properties and health benefits in mammals, and flavonoids are the most abundant polyphenols in plant foods. Previous studies show that higher intakes of flavonoid-rich foods are associated with lower risks of various diseases, including cardiovascular disease and cancer [18]. *A. marina* is the most abundant and dominant mangrove species and has a long history of use as a traditional medicine for the treatment for skin diseases, rheumatism, ulcers, and smallpox. However, few previous studies have characterized the relationship between anticancer activities and polyphenol contents. The present study shows anticancer activities of four different extracts of *A. marina* in three human breast cancer cell lines, two liver human cancer cell lines, and in one normal cell line, and in a mouse xenograft model. Taken together, these data indicate chemotherapeutic potential of *A. marina* extracts and fractions, and the ensuing anticancer efficacies were correlated with total phenol contents. The present findings also show that treatment with *A. marina* leaf extracts and fractions leads to activation of PARP, caspase 3, and caspase 8 in AU565, BT483, and HepG2 cancer cell lines. EtOAc and EtOH extracts inhibited MDA-MB-231 xenograft tumor growth in nude mice. Accordingly, treatment with low-dose (10–20 μg/mL) EtOAc extracts for 24 h decreased cell migration by downregulating protein expression of MMP2, MMP9, cyclin B, vimentin, and snail protein in MDA-MB-231 cells, indicating decreased breast cancer cell growth both *in vivo* and *in vitro*.

Mangroves produce a wide variety of secondary metabolites with demonstrated anticancer properties, including alkaloids, triterpenoids, flavonoids, and tannins. Here EtOAc extracts of *A. marina* leaves had the highest phenol and flavonoid contents, and also had the best anticancer activities. The present F2-5, F3-2-9, and F3-2-10 fractions showed higher cytotoxic effects than the raw EtOAc extracts. In particular, F2-5 and F3-2-9 fractions exhibited strong inhibitory effects on AU565 cells, with IC_50_ values of 0.75 μg/mL and 2.1 μg/mL, respectively.

Because the criteria of the American National Cancer Institute consider IC_50_ values of < 30 μg/mL indicative of a promising extract [19], F2-5 and F3-2-9 can be considered as potential sources of anticancer drugs. However, whether EtOAc extracts of *A. marina* leaves contain other secondary metabolites, such as alkaloids, triterpenoids, and tannins, requires confirmation in further studies.

The present HPLC data showed that F3-2-9 contains two major peaks with retention times close to those of luteolin and quercetin standards. Luteolin is present in a fraction from a MeOH extract of *A. marina* that had high anticancer activity [20]. However, the present EtOAc extracts had higher cytotoxic effects than MeOH extracts. Hence, further structural comparisons of these fractions are warranted to elucidate anticancer mechanisms. Here reversed-phased HPLC of the F3-2-10 fraction yielded avicennones D and E, which were previously isolated from in 2007 by Li et al. [21]. However, corresponding anticancer activities of these compounds remain uncharacterized. Hence, the present data are the first to indicate apoptotic mechanisms of avicennone D and E in HPLC fractions from EtOAc extracts of *A. marina* leaves in breast and liver cancer cells.

In summary, EtOAc extracts of *A. marina* leaves induce apoptosis and inhibit migration of breast and liver cancer cells. These results warrant further investigation of *A. marina* leaf extracts to identify candidate drugs for the treatment of breast and liver cancers, either alone or in combination with other drugs.

## MATERIALS AND METHODS

### Preparation of *A. marina* extracts and ethyl acetate (EtOAc) fractions

Raw *A. marina* materials were collected from the Xinfeng mangrove conservation area in Taiwan. To prepare water (H_2_O) extracts, 100 g of dried *A. marina* leaves or seeds were immersed in 1000 mL of distilled water for 30 min and were then boiled at 95°C for 30 min. After cooling, the mixture was filtered through a filter paper and water extracts were obtained after evaporation to dryness in a vacuum rotary evaporator at 80°C. To prepare methanol (MeOH) and ethanol (EtOH) extracts, 100 g of *A. marina* dried leaves or seeds were immersed in 1000 mL of MeOH or EtOH for 24 h at 4°C and were then filtered using filter papers. Filtrates were then evaporated under vacuum at 55°C. To prepare ethyl acetate (EtOAc) extracts, water extracts were reconstituted in distilled water and were then partitioned sequentially with equal volumes of EtOAc as indicated in Figure 1A. EtOAc soluble fractions were then fractionated using various column chromatography protocols as indicated in Figure 4A. Tested concentrations were expressed according to dry weights of extracts as indicated in Table 1. Furthermore, the chemical structures were identified by NMR spectroscopy.

### Cell viability assays

Cells were treated with *A. marina* extracts for 48 h and cell viability was determined using MTT [3-(4, 5-dimethylthiazol-2-yl)-2,5-diphenyl tetrazolium bromide] assays. Briefly, MTT-formazan crystals were formed in metabolically viable cells and were dissolved in 500 μl of DMSO for spectrophotometric determinations at a wavelength of 550 nm.

### Determinations of total phenol and flavonoid contents

Total phenol contents were determined spectrophotometrically using gallic acid as a standard according to a previously described Folin-Ciocalteu method [22]. Total flavonoid contents were also determined spectrophotometrically using quercetin as a standard, according to a previously reported method [23].

### Determination of trace metal elements in *A. marina* leaves using inductively coupled plasma atomic emission spectroscopy (ICP-AES)

ICP-AES was used for rapid, precise, and accurate determinations of trace metal elements in digested *A. marina* leaves. Briefly, 0.5-g leaf extracts were digested by heating in a sealed teflon microwave digestion vessel (MARS Xpress TFM high pressure reaction vessel) containing 10 mL of concentrated nitric acid. Teflon vessels were then incubated at 180°C for 10 min and after cooling to the room temperature, contents of vessels were thoroughly transferred into 50 mL polypropylene test tubes and were diluted to 10 mL in deionized water. Sample solutions were then filtered and concentrations of trace metal elements were measured directly using ICP-AES (PerkinElmer ICP Optima 21000bv).

### High performance liquid chromatography (HPLC) analyses of *A. marina* extracts

Concentrations of polyphenol compounds in *A. marina* extracts were determined using HPLC with a 250 × 4.6 mm i.d., 5 μm Thermo 5 C18-MS packed column (Nacalei Tesque, Inc., Kyoto, Japan). Gradient elution was performed using mobile phases comprising (A) 15% acetonitrile, 4% ethyl acetate, 0.1% formic acid and 80.9% ddH2O, and (B) 45% acetonitrile, 4% ethyl acetate, 0.1% formic acid and 50.9% ddH2O. The flow rate was 1 mL/min and polyphenols were detected spectrophotometrically at 280 nm.

### Soft agar colony formation assays

Equal volumes of single-cell suspensions were treated with EtOAc extracts of *A. marina* leaves in DMEM containing 20% fetal bovine serum (FBS), and were then mixed with 0.7% agarose. Mixtures were plated on 6-cm culture dishes on top of a base layer of 0.7% agarose containing 10% FBS, and were allowed to gel. After 14–21-days incubation, colonies of > 60 μm were counted using a light microscope.

### Flow cytometry

Flow cytometric analyses were performed as previously described [24], and fluorescent propidium idodide (PI)-DNA complexes were quantified using FAC-Scan cytometry.

### Trypan blue exclusion assays

Trypan blue dye exclusion assays were used to determine cytotoxic effects of *A. marina* leaf extracts in AU565, BT483, and HepG2 cells. Briefly, cells were seeded in six-well plates at a density of 5 × 10^4^ cells in DMEM containing 10% FBS, and were then incubated for 24 h at 37°C. Culture medium was replaced and cells were incubated with or without the indicated concentrations of *A. marina* leaf extracts for 24–72 h in DMEM containing 2% FBS. Cells were then harvested by trypsinization and were mixed with trypan blue solution, and dead stained cells were counted using a hemocytometer under a microscope.

### DNA fragmentation assays

DNA fragmentation assays were performed as previously described [19]. Briefly, cells were incubated with reagents at various concentrations for 48 h and were then lysed using lysis buffer. DNA was then extracted using phenol/chloroform/isoamyl alcohol and fragments were visualized using 2% agarose gel electrophoresis.

### Analyses of nuclear morphology

Cells were plated on coverslips in six-well plates. After treatment with *A. marina* leaf extracts for 48 h, cells were fixed with 4% formaldehyde for 20 min and were then incubated in 4-mg/mL Hoechst 33258 for 30 min. Coverslips were washed and mounted in Vectashield (Vector Laboratories, Burlingame, CA) and nuclear chromatin morphology was viewed under a microscope.

### Western blot analyses

Western blot analyses were performed as previously described [19], and immunoblots were visualized using enhanced chemiluminescent reagent.

### Animals and treatments

Female BALB/c nude mice (6–8 weeks old) were purchased from the National Laboratory Animal Breeding and Research Center (Taipei, Taiwan). A total of 2 × 10^6^ MDA-MB-231 cells were subcutaneously implanted into the legs of female nude mice. Mice were then randomly divided into three groups (four mice per group) for intraperitoneal treatments with DMSO, EtOH (200 mg/kg/day), and EtOAc extracts (200 mg/kg/day) for 23 days. Tumor volumes were calculated using the following formula: Volume (mm^3^) = A × B^2^/2, where A is the longest diameter (mm) and B is the shortest diameter (mm).

### Statistical analyses

All data are expressed as means ± standard errors of the mean (SE) of at least three separate experiments for each group. Differences were identified using Student's *t*-tests and were considered significant when *p < 0.05, **p < 0.01, or ***p < 0.001.
